# FPGA Implementation of Generalized Hebbian Algorithm for Texture Classification

**DOI:** 10.3390/s120506244

**Published:** 2012-05-10

**Authors:** Shiow-Jyu Lin, Wen-Jyi Hwang, Wei-Hao Lee

**Affiliations:** 1 Department of Electronic Engineering, National Ilan University, Yilan 260, Taiwan; E-Mail: sjlin@niu.edu.tw; 2 Department of Computer Science and Information Engineering, National Taiwan Normal University, Taipei 116, Taiwan; E-Mail: 699470125@ntnu.edu.tw

**Keywords:** system on programmable chip, reconfigurable computing, principal component analysis, generalized Hebbian algorithm, texture classification, FPGA

## Abstract

This paper presents a novel hardware architecture for principal component analysis. The architecture is based on the Generalized Hebbian Algorithm (GHA) because of its simplicity and effectiveness. The architecture is separated into three portions: the weight vector updating unit, the principal computation unit and the memory unit. In the weight vector updating unit, the computation of different synaptic weight vectors shares the same circuit for reducing the area costs. To show the effectiveness of the circuit, a texture classification system based on the proposed architecture is physically implemented by Field Programmable Gate Array (FPGA). It is embedded in a System-On-Programmable-Chip (SOPC) platform for performance measurement. Experimental results show that the proposed architecture is an efficient design for attaining both high speed performance and low area costs.

## 1. Introduction

Principal Component Analysis (PCA) [1] plays an important role in pattern recognition, classification, computer vision and data compression [2,3]. It is an effective feature extraction technique capable of finding a compact and accurate representation of the data that reduces or eliminates statistically redundant components. Basic PCA implementation involves the Eigen-Value Decomposition (EVD) of the covariance matrix. Long computation time and large storage size are usually required for the EVD. The basic PCA therefore is not suited for online computation on the platforms with limited computation capacity and storage size.

To compute the PCA with reduced computational complexity, a number of fast algorithms [2,4–6] have been proposed. The algorithm presented in [4] is based on Expectation Maximization (EM). The inverse matrix computation is required in the algorithm, which may be an expensive exercise. Incremental and/or iterative algorithms for PCA computations are proposed in [2,5,6]. A common drawback of these fast PCA methods is that the covariance matrix of training data should be involved. The computation time and storage may still be expensive. Although hardware implementation of PCA is possible, large storage size and complicated circuit control management are usually necessary. The PCA hardware implementation therefore may be used only for data with small dimensions [7–9] when limited hardware resource is available. Because of the difficulties for hardware implementation, many PCA-based applications use software for the PCA computation. After the eigenvectors are obtained, only the projection computation is implemented by hardware [10–12].

An alternative for the PCA implementation is to use the Generalized Hebbian Algorithm (GHA) [13,14]. The GHA is based on an effective incremental updating scheme without the involvement of covariance matrix. The storage requirement for the PCA implementation is then significantly reduced. Nevertheless, slow convergence of the GHA is usually observed. A large number of iterations therefore is required, resulting in long computational time. An effective approach to expedite the GHA training is based on multithreading techniques, which take advantages of all the cores of multicore processors to reduce the computational time. However, multicore processors usually consume large power [15], and therefore may not be suited for applications requiring low power dissipation.

Analog hardware implementations of GHA [16,17] have been found to be a power efficient approach for accelerating the computational speed. However, these architectures are difficult to be directly used for digital devices. A number of digital hardware architectures [18,19] have been proposed for expediting the GHA training process. The architecture in [18] separates the weight vector updating process of GHA into a number of stages for data reuse. Although the architecture has fast computation time, its hardware resource utilization grows linearly with the dimension of data and number of principal components. Therefore, the architecture may not be well suited for data with high vector dimension and/or large number of principal components.

A systolic array with low area costs is proposed in [19]. The systolic array is based on pixel-wise operations so that the area costs for weight vector updating are independent of vector dimension. Nevertheless, the latency of the architecture increases with the dimension of data. Moreover, similar to the architecture in [18], the area costs of [19] grow with the number of principal components. Therefore, the architecture may still have long latency and high area costs.

In light of the facts stated above, a novel GHA implementation capable of performing fast PCA with low power consumption is presented. The implementation is based on Field Programmable Gate Array (FPGA) because it consumes lower power over its multicore counterparts [20,21]. As compared with existing FPGA-based architectures for GHA, the proposed architecture has lower area cost and/or lower latency. The proposed architecture can be divided into three parts: the Synaptic Weight Updating (SWU) unit, the Principal Components Computing (PCC) unit, and the memory unit. The memory unit is the on-chip memory storing training vectors and synaptic weight vectors. Based on the data stored in the memory unit, the SWU and PCC units are then used to compute the principal components and update the synaptic weight vectors, respectively.

In the SWU and PCC units, the input training vectors and synaptic weight vectors are separated into a number of non-overlapping blocks for principal component computation and synaptic weight vector updating. Both the SWU and PCC units operate one block at a time. In each unit, the operations of different blocks share the same circuit for reducing the area costs. Moreover, in the SWU unit, the results of precedent weight vectors will be used for the computation of subsequent weight vectors for reducing training time.

To demonstrate the effectiveness of the proposed architecture, a texture classification system on a System-On-Programmable-Chip (SOPC) platform is constructed. The system consists of the proposed architecture, a softcore NIOS II processor [22], a DMA controller, and a SDRAM. The proposed architecture is adopted for finding the PCA transform by the GHA training, where the training vectors are stored in the SDRAM. The DMA controller is used for the DMA delivery of the training vectors. The softcore processor is only used for coordinating the SOPC system. It does not participate the GHA training process. As compared with its multithreaded software counterpart running on Intel multicore processors, our system has lower computational time and lower power consumption for large training set. All these facts demonstrate the effectiveness of the proposed architecture.

### 2. Preliminaries

**Figure 1.**
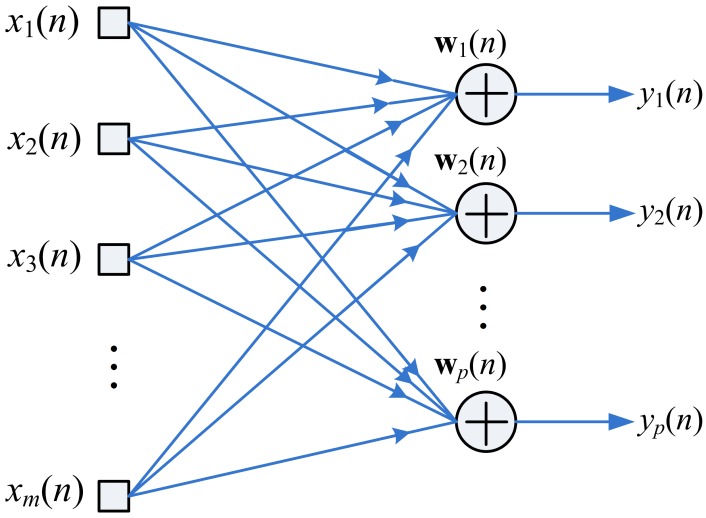
The neural model for the GHA.

Figure 1 shows the neural model for GHA, where x(*n*) = [*x*_1_(*n*),…,*x_m_*(*n*)]*^T^*, and y(*n*) = [*y*_1_(*n*), …,*y_p_*(*n*)]*^T^* are the input and output vectors to the GHA model, respectively. In addition, *m* and *p* are the vector dimension and the number of Principal Components (PCs) for the GHA, respectively. The output vector y(*n*) is related to the input vector x(*n*) by

(1) $$y_{i}(n)=\sum_{i=1}^{m}w_{ji}(n)x_{i}(n)$$

where the *w_ji_*(*n*) stands for the weight from the *i*-th synapse to the *j*-th neuron at iteration *n*.

Let

(2) $$w_{j}(n)={\left[w_{j1}(n),\ldots w_{jm}(n)\right]}^{T},j=1,\ldots ,p$$

be the *j*-th synaptic weight vector. Each synaptic weight vector w*_j_*(*n*) is adapted by the Hebbian learning rule:

(3) $$w_{ji}(n+1)=w_{ji}(n)+\eta [y_{i}(n)x_{i}(n)-y_{i}(n)\sum_{k=1}^{j}w_{ki}(n)y_{k}(n)]$$

where *η* denotes the learning rate. After a large number of iterative computation and adaptation, w*_j_*(*n*) will asymptotically approach to the eigenvector associated with the *j*-th eigenvalue λ*j* of the covariance matrix of input vectors, where λ_1_ > λ_2_ > … > λ*_p_*. To reduce the complexity of computing implementation, Equation (3) can be rewritten as

(4) $$w_{ji}(n+1)=w_{ji}(n)+\eta y_{j}(n)[x_{i}(n)-\sum_{k=1}^{j}w_{ki}(n)y_{k}(n)]$$

A more detailed discussion of GHA can be found in [13,14]

## 3. The Proposed GHA Architecture

**Figure 2.**
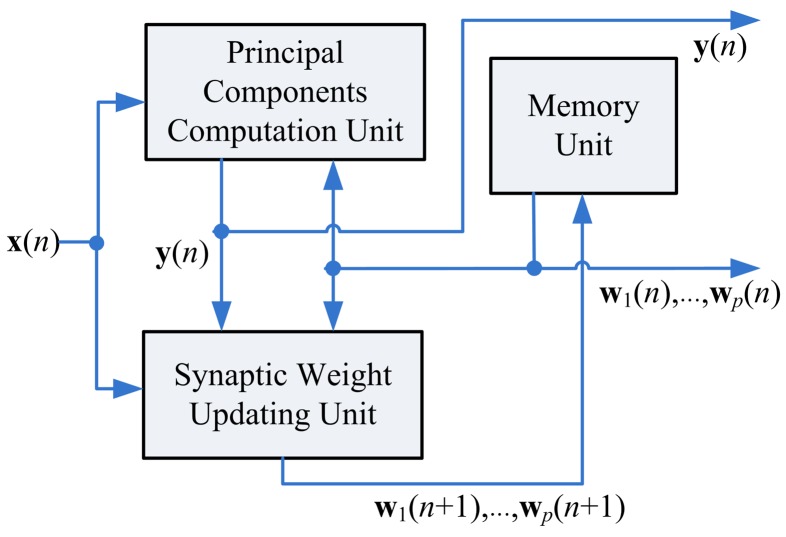
The proposed GHA architecture.

As shown in Figure 2, the proposed GHA architecture consists of three functional units: the memory unit, the Synaptic Weight Updating (SWU) unit, and the Principal Components Computing (PCC) unit. The memory unit is used for storing the *current* synaptic weight vectors and input vectors. Assume the *current* synaptic weight vectors w*_j_*(*n*),*j* = 1,…,*p*, are now stored in the memory unit. In addition, the input vector x(*n*) is available. Based on x(*n*) and w*_j_*(*n*),*j* = 1,…,*p*, the goal of PCC unit is to compute output vector y (*n*). Using x(*n*), y (*n*) and w_j_(*n*),*j =* 1,*…,p*, the SWU unit produces the new synaptic weight vectors w*_j_*(*n* + 1), *j* = 1,…,*p*. It can be observed from Figure 2 that the new synaptic weight vectors will be stored back to the memory unit for subsequent training.

### 3.1. SWU Unit

The design of SWU unit is based on Equation (4). Although the direct implementation of Equation (4) is possible, it will consume large hardware resources. To further elaborate this fact, we first see from Equation (4) that the computation of *w_ji_*(*n* + 1) and *w_ri_*(*n* + 1) shares the same term $\begin{array}{l} \sum_{k=1}^{r}w_{ki}(n)y_{k}(n) \end{array}$ when *r* ≤ *j*. Consequently, independent implementation of *w_ji_*(*n+* 1) and *w_ri_*(*n*+1) by hardware using Equation (4) will result in large hardware resource overhead.

To reduce the resource consumption, we first define a vector *z_ji_*(*n*) as

(5) $$z_{ji}(n)=x_{i}(n)-\sum_{k=1}^{j}w_{ki}(n)y_{k}(n),j=1,\ldots ,p$$

and *z_j_*(*n*) = [*z_j_*_1_(*n*), …, *z_jm_*(*n*)]*^T^*. Integrating Equation (4) and (5), we obtain

(6) $$w_{ji}(n+1)=w_{ji}(n)+\eta y_{j}(n)z_{ji}(n)$$

where *z_ji_*(*n*) can be obtained from *z*_(_*_j−_*_1)_*_i_*(*n*) by

(7) $$z_{ji}(n)=z_{(j-1)i}(n)-w_{ji}(n)y_{j}(n),j=2,\ldots ,p$$

When *j* = 1, from Equations (5) and (7), it follows that

(8) $$z_{0i}(n)=x_{i}(n)$$

**Figure 3.**
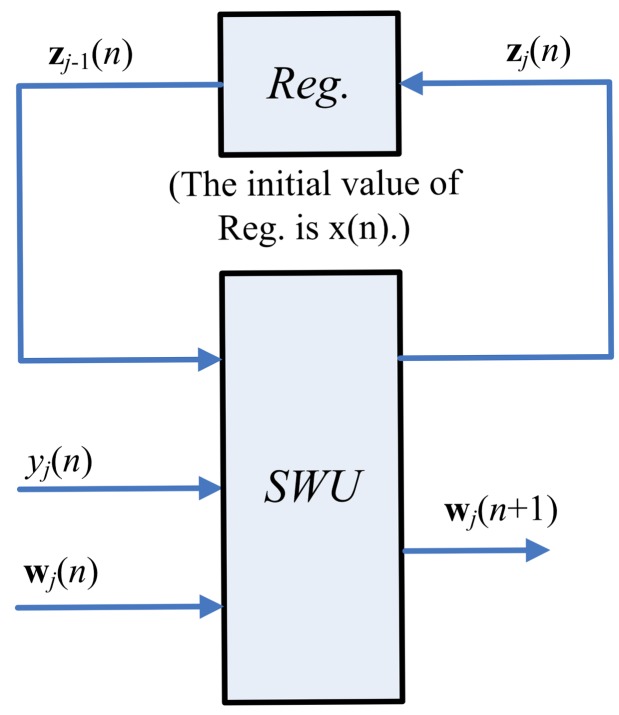
The hardware implementation of Equations (6) and (7).

Figure 3 depicts the hardware implementation of Equations (6) and (7). As shown in the figure, the SWU unit produces one synaptic weight vector at atime. The computation of w_j_(*n +* 1), the *j*-th weight vector at the iteration *n*+1, requires the z*_j_*_−1_(*n*), y(*n*) and w*_j_*(*n*) as inputs. In addition to w_j_(*n*+ 1), the SWU unit also produces *z_j_*(*n*), which will then be used for the computation of w*_j_*_+1_(*n* + 1). Hardware resource consumption can then be effectively reduced.

**Figure 4.**
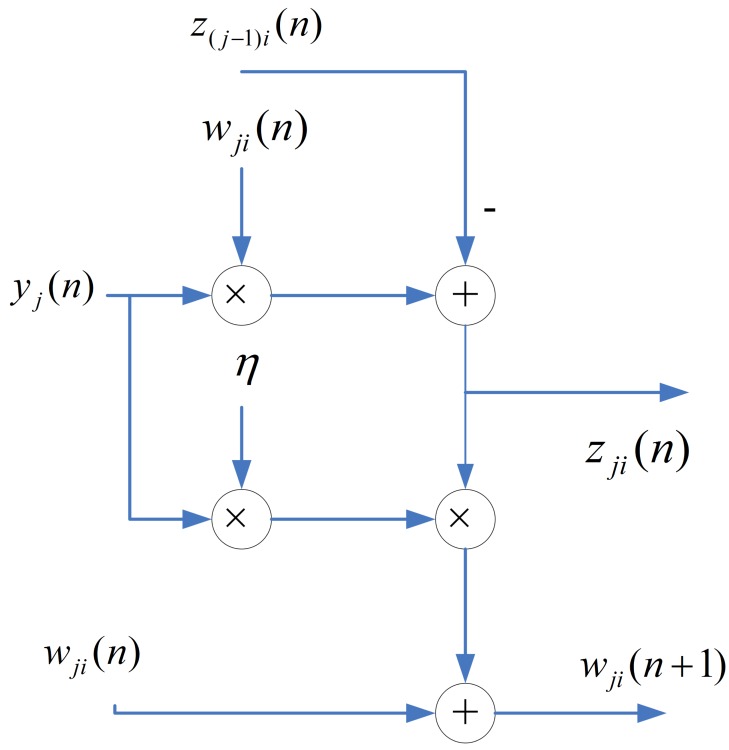
The architecture of each module in the SWU unit.

One way to implement the SWU unit is to produce w*_j_*(*n* + 1) and z*_j_*(*n*) in one shot. However, *m* identical modules, individually shown in Figure 4, may be required because the dimension of vectors is *m*. The area costs of the SWU unit then grow linearly with *m*. To further reduce the area costs, each of the output vectors w*_j_*(*n* + 1) and z*_j_*(*n*) is separated into *b* blocks, where each block contains *q* elements. The SWU unit only computes one block of w*_j_*(*n* + 1) and z*_j_*(*n*) at a time. Therefore, it will take *b* clock cycles to produce complete w*_j_*(*n* + 1) and z*_j_*(*n*).

Let

(9) $${\hat{\text{w}}}_{j,k}(n)={\left[w_{j,(k-1)q+1}(n),\ldots ,w_{j,(k-1)q+q}(n)\right]}^{T},k=1,\ldots ,b$$

and

(10) $${\hat{\text{z}}}_{j,k}(n)={\left[z_{j,(k-1)q+1}(n),\ldots ,z_{j,(k-1)q+q}(n)\right]}^{T},k=1,\ldots ,b$$

be the *k*-th block of w*_j_*(*n*) and z*_j_*(*n*), respectively. The computation w*_j_*(*n* + 1) and z*_j_*(*n*) take *b* clock cycles. At the k-th clock cycle, *k =* 1,…, *b*, the SWU unit computes ŵ*_j,k_*(*n* + 1) and ẑ *_j,k_*(*n*). Because each of ŵ*_j,k_*(*n* + 1) and ẑ*_j,k_*(*n*) contains only *q* elements, the SWU unit consists of *q* identical modules. The architecture of each module is also shown in Figure 4. The SWU unit can be used for GHA with different vector dimension *m*. As *m* increases, the area costs therefore remain the same at the expense of a larger number of clock cycles *b* for the computation of ŵ*_j,k_*(*n* + 1) and ẑ*_j,k_*(*n*).

Based on Equation (8), the input vector z_0_(*n*) is actually the training vector x(*n*), which is also separated into *b* blocks, where the *k*-th block is given by

(11) $${\hat{\text{z}}}_{0,k}(n)={\left[x_{(k-1)q+1}(n),\ldots ,x_{(k-1)q+q}(n)\right]}^{T},k=1,\ldots ,b$$

**Figure 5.**
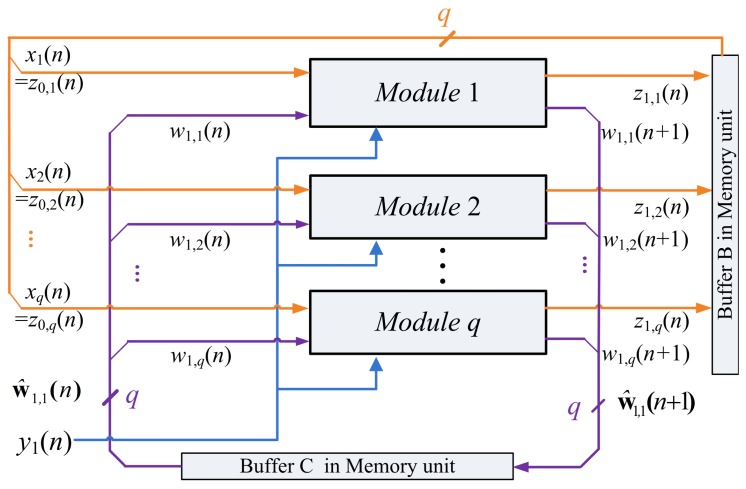
The SWU unit operation for computing the first segment of w_1_(*n*+ 1).

The ẑ_0_*_,k_*(*n*) and ŵ_1,*k*_(*n*), *k* = 1,…, *b*, are used as the input vectors for the computation of ẑ_1,_*_k_*(*n*) and ŵ_1,*k*_(*n* + 1), *k* = 1,…,*b*. The z_1_(*n*) and w_1_(*n* + 1) become available when all the ẑ_1,_*_k_*(*n*) and ŵ_1_*_,k_*(*n* + 1), *k* = 1,…,*b*, are obtained. Figure 5 shows the computation of ẑ_1,1_(*n*) and ŵ_1,1_(*n* + 1) based on ẑ_0,1_(*n*) and ŵ_1,1_(*n*).

After the computation of w_1_(*n* + 1) and z_1_(*n*) are completed, the vector z_1_(*n*) is then used for the computation of z_2_(*n*) and w_2_(*n* +1). The vector z_2_(*n*) is then used for the computation of w_3_(*n* + 1). The weight vector updating process at the iteration *n* + 1 will not be completed until the SWU unit produces the weight vector w*_p_*(*n* + 1).

### 3.2. PCC Unit

The PCC operations are based on Equation (1). Therefore, the PCC unit of the proposed architecture contains adders and multipliers. Because the number of multipliers grows with the vector dimension *m*, the direct implementation using Equation (1) may consume large hardware resources when *m* becomes large. Similar to the SWU unit, the block based computation is used for reducing the area costs. Based on Equations (9) and (11), the Equation (1) can be rewritten as

(12) $$y_{j}(n)=\sum_{k=1}^{b}\sum_{i=1}^{q}w_{j,(k-1)q+i}(n)x_{(k-1)q+i}(n),=\sum_{k=1}^{b}{\hat{\text{w}}}_{j,k}^{T}(n){\hat{\text{z}}}_{0,k}(n)$$

**Figure 6.**
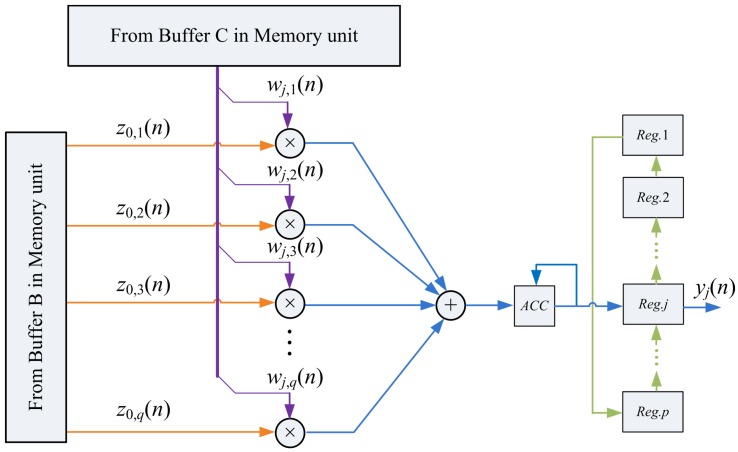
The PCC unit architecture.

The implementation of Equation (12) needs only *q* multipliers, a *q*-input adder, an accumulator, and a *p*-entry buffer, as shown in Figure 6. The multipliers and the *q*-input adder are organized as a *s*-stage pipeline for enhancing the throughput of the circuit.

The blocks ŵ*_j,k_*(*n*) and ẑ_0_*_,k_*(*n*) are the inputs to the PCC unit. Figure 6 also shows the operation of PCC unit when the input vectors are ŵ*_j,_*_1_(*n*) and ẑ_0,1_(*n*). Note that the output of the accumulator in the circuit becomes *y_j_*(*n*) only after all the blocks ŵ*_j_*_,k_(*n*) and ẑ_0,_*_k_*(*n*), *k =* 1,…,*b*, have been fetched from the memory unit. The computation of each *y_j_*(*n*) therefore takes *b* + *s* cycles. After the computation of *y_j_*(*n*) is completed, *y_j_*(*n*) will be stored in the *j*-th entry of the buffer for the subsequent computation of w*_j_*(*n* + 1) in the SWU unit.

### 3.3. Memory Unit

The memory unit contains three buffers: Buffer A, Buffer B and Buffer C. Buffer A fetches and stores training vector x(*n*) from the main memory. Buffer B contains z*_j_*(*n*) for the computation in PCC and SWU units. The synaptic weight vectors w*_j_*(*n*) are stored in Buffer C. All the buffers are shift registers.

**Figure 7.**
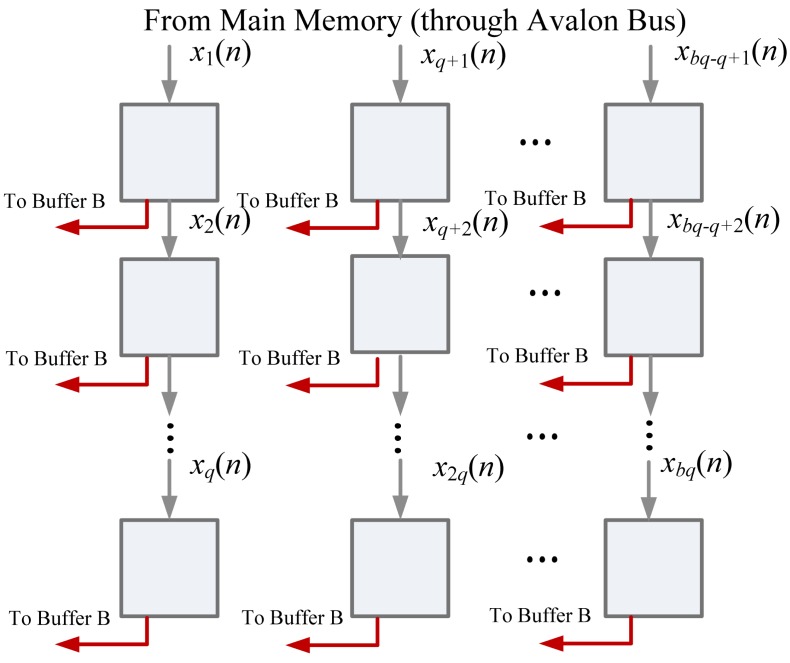
The Buffer A architecture in memory unit.

To fetch training vector x(*n*) from main memory, the *m* elements in the training vector are interleaved and separated into *q* segments. Each segment contains *b* elements. Therefore, Buffer A is a *q*-stage shift register, where each stage contains *b* cells, as shown in Figure 7. Upon all the *q* segments are received, they are copied to Buffer B as z_0_(*n*).

**Figure 8.**
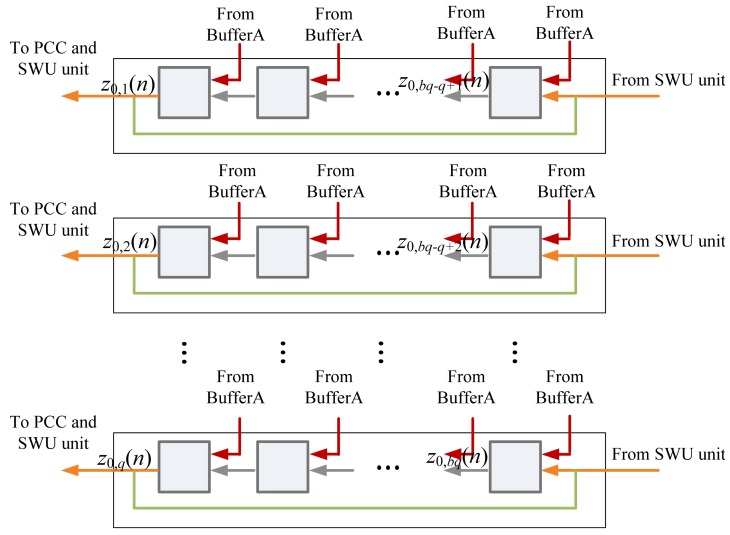
The Buffer B architecture in memory unit.

**Figure 9.**
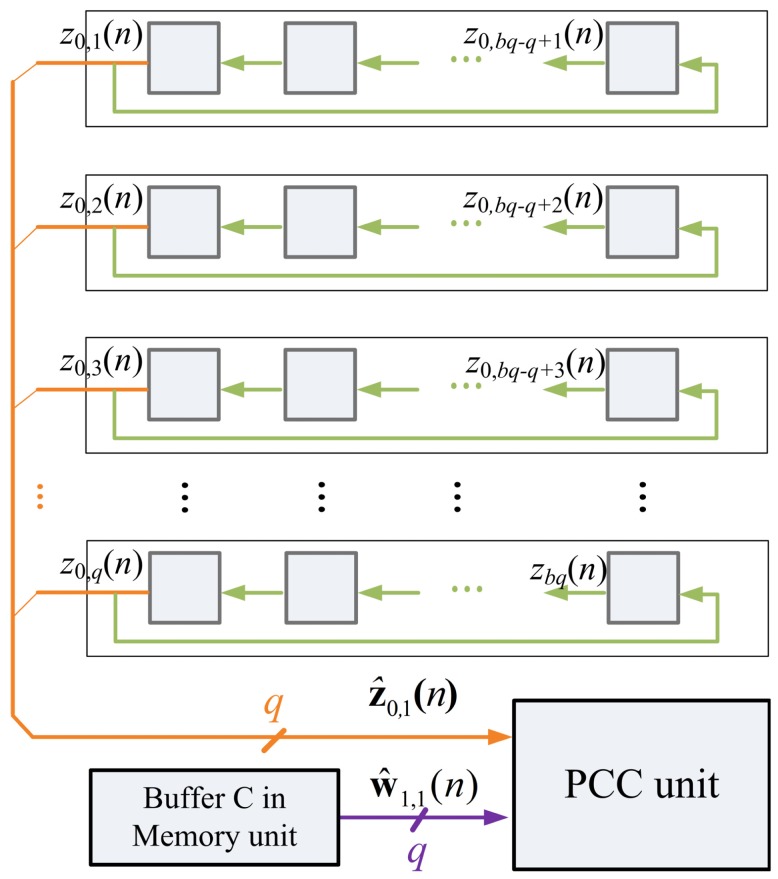
The Buffer B operation for the PCC unit.

The architecture of Buffer B is depicted in Figure 8. It holds the values of z*_j_*(*n*) for the computation in PCC and SWU units. The data in Buffer B is initialized by Buffer A. That is, the initial content of Buffer B is x(*n*) (*i.e.*, z_0_(*n*)). As shown in Figure 9, Buffer B then provides *b* blocks ẑ_0_*_,k_*(*n*), *k* = 1,…,*b*, sequentially to PCC unit for the computation of *y_j_*(*n*). Because z_0_(*n*) are used for the operations in PCC and SWU units, all the data output to PCC unit is also rotated back to Buffer B.

**Figure 10.**
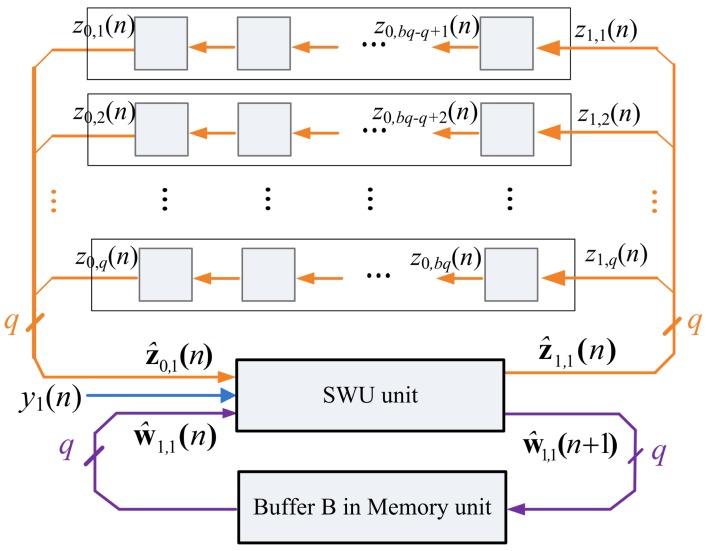
The Buffer B operation for the SWU unit.

After the PCC computation is completed, the Buffer B then delivers data for SWU unit. Starting from z_0_(*n*), the Buffer B provides z*_j_*(*n*) to SWU unit, and then receives z_*j*+1_(*n*) from SWU unit for j = 0,…, *p* − 1. The delivery of z*_j_*(*n*) and collection of z_*j*+1_(*n*) are on a block-by-block basis, as depicted in Figure 10.

**Figure 11.**
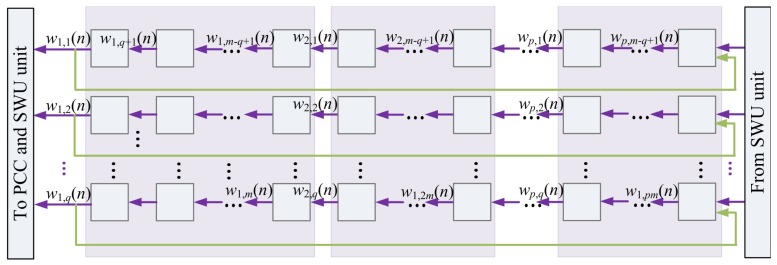
The Buffer C architecture.

**Figure 12.**
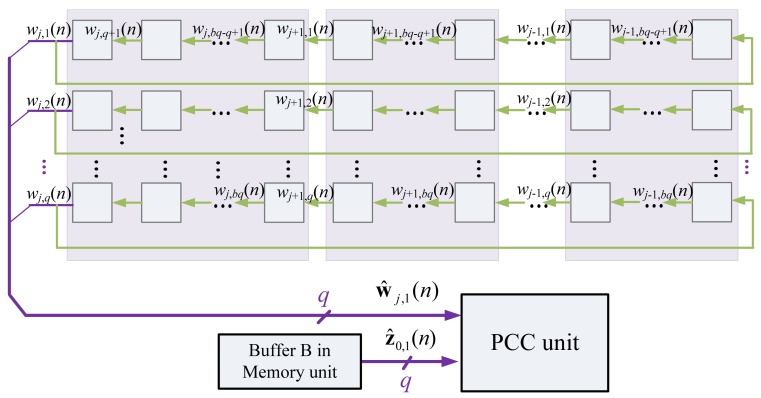
The Buffer C operation for the PCC unit.

The Buffer C contains the synaptic weight vectors w_j_(*n*), j = 1,…,*p*. In addition to providing and storing data for the computation in PCC and SWU units, it also holds the final results after GHA training. Figure 11 shows the architecture of Buffer C. Similar to Buffer B, each synaptic weight vectors w_j_(*n*) is divided into b blocks. They are delivered to PCC unit sequentially for the computation of *y_j_*(*n*). Moreover, since w*_j_*(*n*) is also needed for the computation of w_j_(*n* + 1) in the SWU unit, the *b* blocks delivered to the PCC unit should also be rotated back to Buffer C. Figure 12 shows the operation of Buffer C for computation in PCC unit.

**Figure 13.**
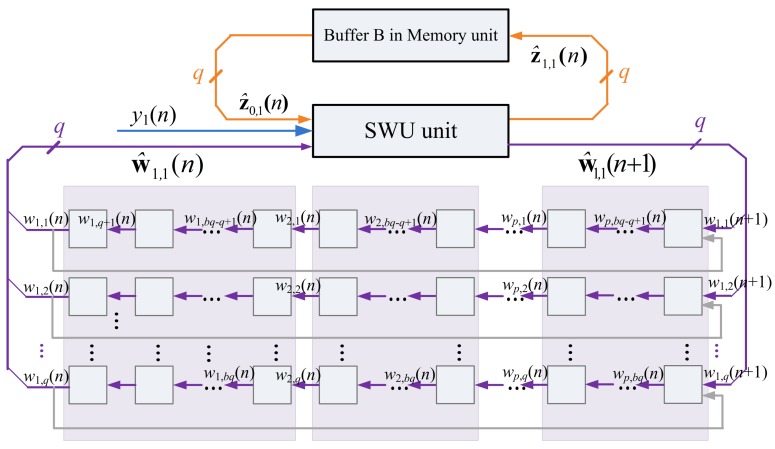
The Buffer C operation for the SWU unit.

To support the computation in SWU unit, the Buffer C delivers w*_j_*(*n*) to SWU unit,and then receives w*_j_*(*n*+1) from the unit. The delivery of w*_j_*(*n*) and collection of w*_j_*(*n*+1) are also on a block-by-block basis, as depicted in Figure 13.

**Figure 14.**
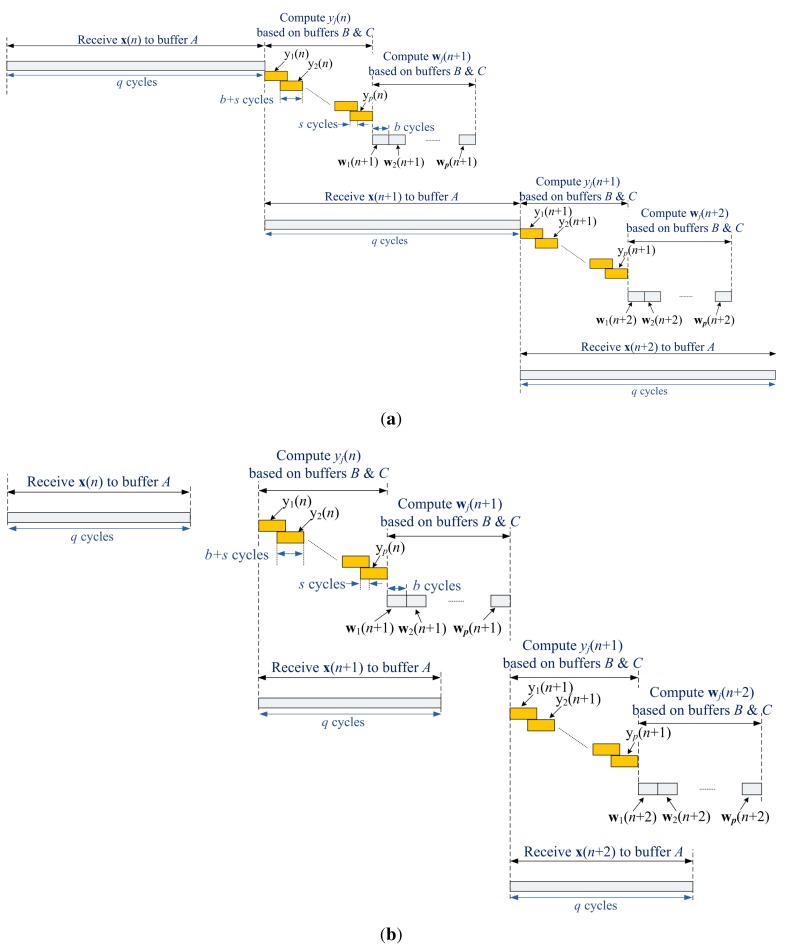
The timing diagram for the operations of the proposed architecture: (**a**) *q* > 2*bp* + *s*; (**b**) *q* < 2*bp* + *s*.

Based on the operations of the memory unit, Figure 14 shows the timing diagram of the proposed architecture. It can be observed from the figure that the Buffer A is operated concurrently with Buffers B and C. That is, while the proposed architecture is fetching the training vector x(*n* + 1) to Buffer A, it is also computing y*_j_*(*n*) and *w_j_*(*n*+1) based on x(*n*) and w(*n*). Fetching training vectors may be a time consuming process as vector dimension grows. Therefore, parallel operations of training vector fetching and weight vector computation are beneficial for increasing the GHA training speed.

### 3.4. SOPC-Based GHA Training System

**Figure 15.**
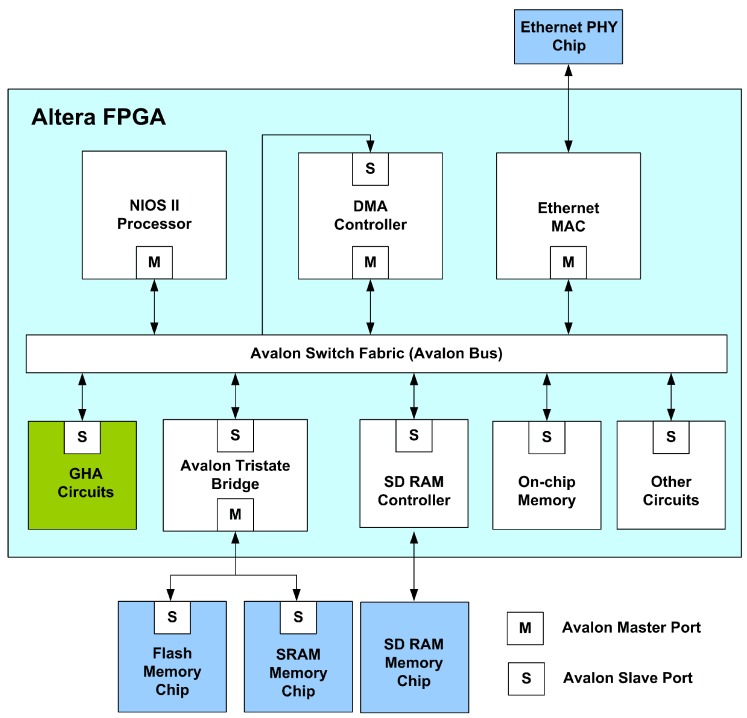
The SOPC system for implementing GHA.

The proposed architecture is used as a custom user logic in a SOPC system consisting of softcore NIOS CPU [22], DMA controller and SDRAM, as depicted in Figure 15. All training vectors are stored in the SDRAM and then transported to the proposed circuit via the Avalon bus. The DMA-based training data delivery is performed so that the memory access overhead can be minimized. The softcore NIOS CPU runs on a simple software to support the proposed circuit for GHA training. The software is used only for coordinating different components in the SOPC platform. It does not involve GHA computations. As the delivery of the training vectors is completed, the softcore CPU then retrieves the training results from proposed architecture for subsequent classification operations.

**Figure 16.**
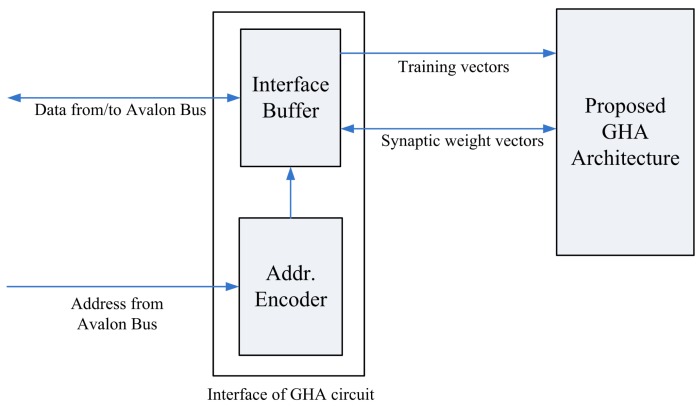
The interface of the proposed architecture to the SOPC system.

**Figure 17.**
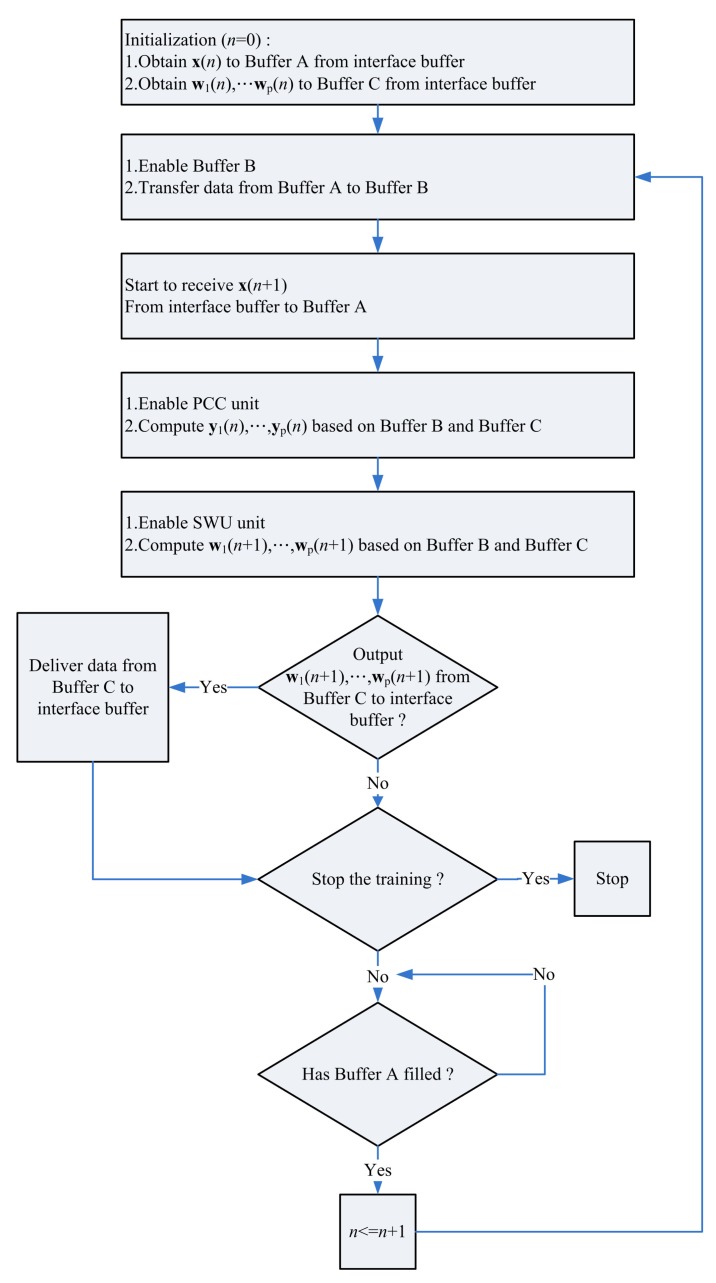
The operation of the controller of the proposed architecture.

Figure 16 depicts the interface of the proposed architecture to the SOPC system. The interface consists of an interface buffer for transferring data between the proposed GHA architecture and the SOPC system. The proposed GHA architecture contains a simple controller for accessing the interface. Figure 17 depicts the operations of the controller. As shown in Figure 17, the proposed circuit fetches the training vectors from the interface buffer to Buffer A for subsequent processing. In addition, after the completion of training, the synaptic weight vectors in Buffer C are delivered to the interface buffer so that they can be accessed by the NIOS CPU.

## 4. Performance Analysis and Experimental Results

The area complexities and latency are the major performances considered in this study. Because adders, multipliers and registers are the basic building blocks of the GHA architecture, the area complexities are separated into three categories: the number of adders, the number of multipliers and the number of registers. Given the current synaptic weight vectors w*_j_*(*n*), *j* = 1,…,*p*, the latency of the proposed GHA architecture is defined as the time required to produce the new synaptic weight vectors w*_j_*(*n* + 1), j = 1,…,*p*.

Table 1 shows the area complexities and latency of various architectures for GHA training. It can be observed from the table that the number of adders and multipliers of the proposed architecture are independent of the vector dimension *m* and the number of principal components *p*. By contrast, the area costs of [18] grow with both *m* and *p*. We can also see from the table that the latency of [19] increases with both *m* and *p*. Based on the timing diagram shown in Figure 14, the latency of the proposed architecture is *max*(*q*, 2*bp* + *s*). Therefore, it is independent of vector dimension *m*. The proposed architecture is then well suited for applications requiring large vector dimension *m*.

Next we consider the physical implementation of the proposed architecture. The design platform is Altera Quartus II with SOPC Builder [23] and NIOS II IDE. Table 2 show the hardware resource consumption of the proposed architecture for vector dimensions *m* = 16 × 16 and *m* = 32 × 32, respectively. The hardware resource utilization of the entire SOPC systems is revealed in Table 3. In order to maintain low area cost, we use fixed-point format to represent data. The length of the format is signed 8 bits. The target FPGA device is Altera Cyclone IV EP4CGX150DF31C7. The number of modules *q* is 64 for all the implementations shown in the tables.

Three different area resources are considered in the tables: Logic Elements (LEs), embedded memory bits, and embedded multipliers. The LEs are used for the implementation of adders, multipliers and registers in the proposed GHA architecture. Both the LEs and embedded memory bits are also used for the implementation of NIOS CPU of the SOPC system. The embedded multipliers are used for the implementation of the multipliers of the proposed GHA architecture.

It can be observed from Tables 2 and 3 that the consumption of embedded multiplier of the proposed architecture is independent of the vector dimension *m* and number of principal components *p*. Because the embedded multipliers are used only for the implementation of multiplier in the proposed architecture, they are dependent only on *q*. In the experiment, all the implementations in Tables 2 and 3 have the same *q*. Therefore, all the implementations utilize the same number of embedded multipliers.

Because the embedded memory bits are mainly used only for the realization of NIOS CPU, the consumption of embedded memory bits are also independent of *m* and *p*, as shown in Tables 2 and 3. It can be observed from the tables that the consumption of LEs grows with *m* and *p*. It is not surprising because the LEs are used to design the registers. Moreover, the number of registers increases with m and *p*, as shown in Table 1. Therefore, the numerical results shown in Tables 2 and 3 are consistent with the analytical results in Table 1.

**Figure 18.**
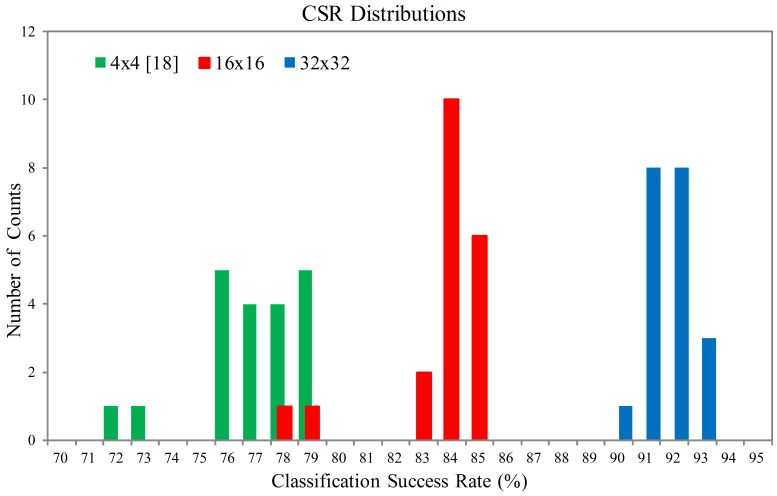
The CSR distributions of the proposed architecture for the texture set shown in Figure 20.

**Figure 19.**
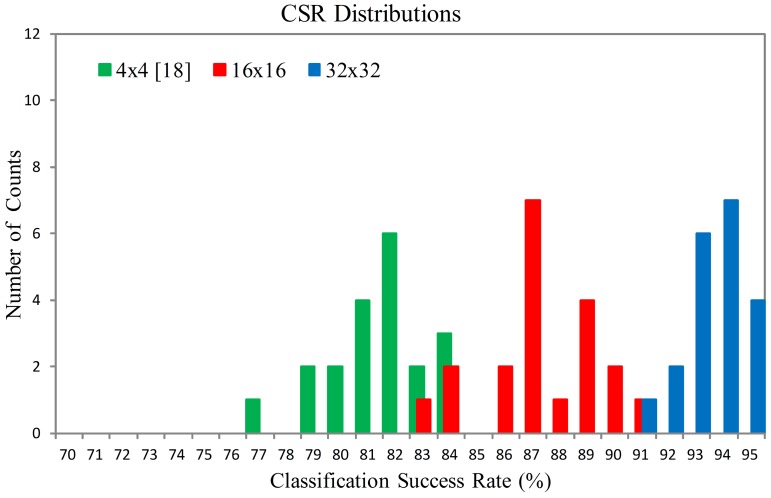
The CSR distributions of the proposed architecture for the texture set shown in Figure 21.

**Figure 20.**
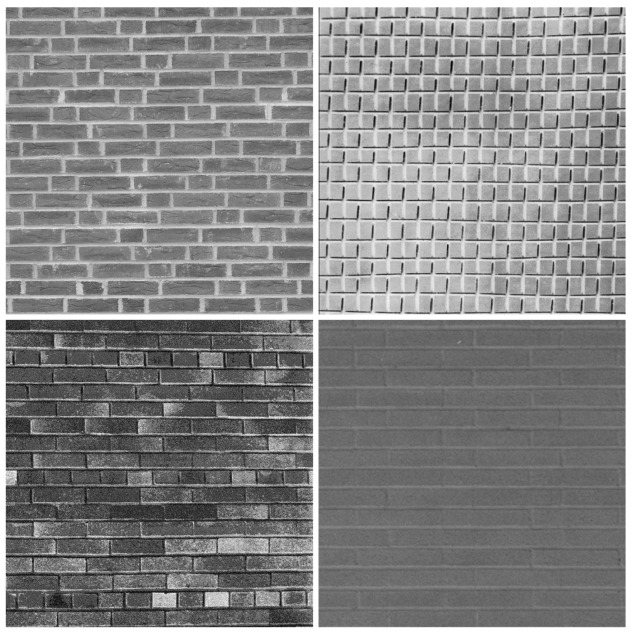
The set of textures for CSR measurements in Figure 18.

**Figure 21.**
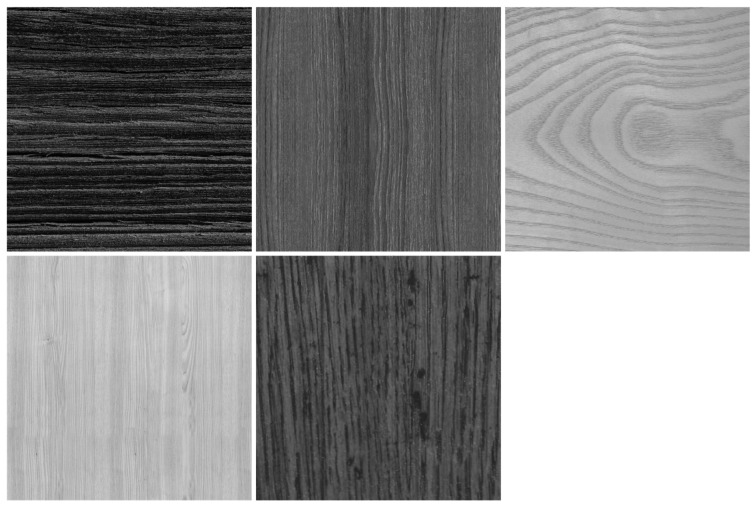
The set of textures for CSR measurements in Figure 19.

Figures 18 and 19 show the Classification Success Rate (CSR) distribution of the proposed architecture for the textures shown in Figures 20 and 21, respectively. The CSR is defined as the number of test vectors which are successfully classified divided by the total number of test vectors. The number of principal components is *p* = 4. The vector dimensions are *m* = 16×16 and 32×32. The distribution for each vector dimension is based on 20 independent GHA training processes. The CSR distribution of the architecture presented in [18] with the same *p* is also included for comparison purpose. The vector dimension for [18] is *m* = 4 × 4.

The size of each texture in Figures 20 and 21 is 576×576. In the experiment, the Principal Component based *k* Nearest Neighbor (PC-*k*NN) rule is adopted for texture classification. Two steps are involved in the PC-*k*NN rule. In the first step, the GHA is applied to the input vectors to transform *m* dimensional data into *p* principal components. The synaptic weight vectors after the convergence of GHA training are adopted to span the linear transformation matrix. In the second step, the *k*NN method is applied to the principal subspace for texture classification.

It can be observed from Figures 18 and 19 that the proposed architecture has better CSR. This is because the vector dimensions of the proposed architecture are higher than those in [18]. Spatial information of textures therefore can be effectively exploited. The proposed architecture is able to implement the hardware GHA training with vector dimension up to *m* = 32 × 32. The hardware realization for *m* = 32 × 32 is possible because the area costs of the SWU and PCC units in the proposed architecture are independent of vector dimension. By contrast, the area costs of the SWU and PCC units in [18] grow with the vector dimension. Therefore, only smaller vector dimension (*i.e., m* = 4 × 4) can be implemented.

**Figure 22.**
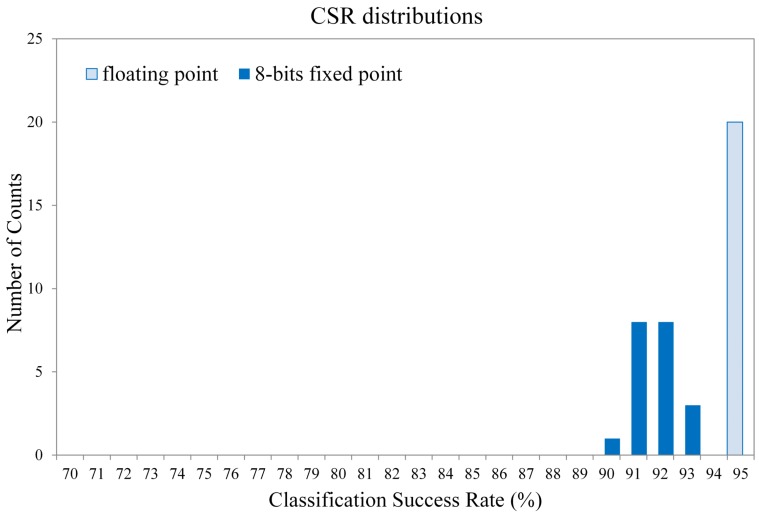
The CSR distribution of GHA with fixed and floating point format.

Although the proposed architecture is based on signed 8-bit fixed point format, the degradation in CSR is small as compared with the GHA without truncation. Figure 22 reveals the truncation effects of the proposed architecture. The GHA implementation without truncation is implemented by software with floating-point format. The training images for this experiment is shown in Figure 20. The vector dimension is 32 × 32. The distribution for each format is based on 20 independent GHA training processes. It can be observed from Figure 22 that only a slight decrease in CSR is observed for the fixed-point format. In fact, the average CSR degradation is only 3.44% (from average CSR 95.53% for floating-point format to 92.09% for fixed point format).

**Figure 23.**
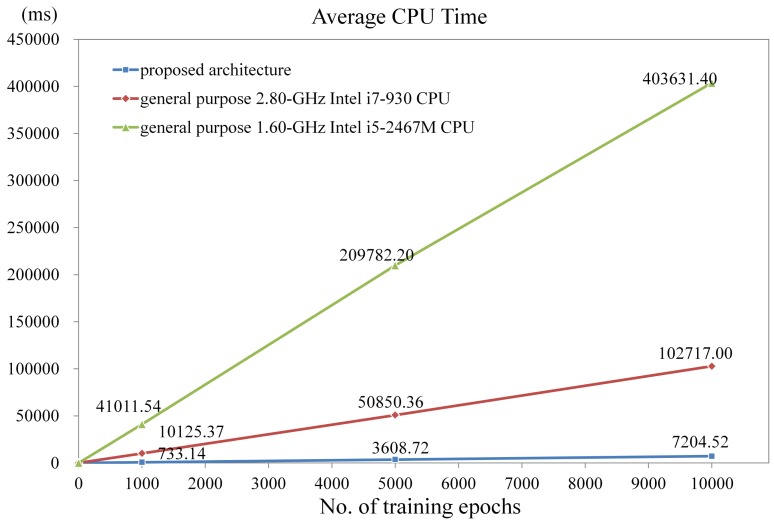
The CPU time of the NIOS-based SOPC system using the proposed architecture as the hardware accelerator for various numbers of training iterations with *m* = 16 × 16 and *p* = 7.

Another advantage of the proposed architecture is its superior computational capacity for GHA training. Figure 23 shows the CPU time of the NIOS-based SOPC system using the proposed architecture as a hardware accelerator for various numbers of training iterations with *m* = 16 × 16 and *p* = 7. The NIOS CPU clock rate in the system is 50 MHz. The target FPGA for the implementation is Cyclone III EP3C120F780C8. The CPU time of the software counterparts running on the general purpose 1.6 GHz Intel i5 and 2.8 GHz Intel i7 processors also are depicted in the Figure 23 for comparison purpose. The software implementations are multithreaded to take advantages of all the cores in the processors. There are 16 threads in the codes: 8 threads for synaptic weight updating, and 8 threads for the principal component computation and others. An optimizing compiler (offered by Visual Studio) is used to further enhance the computational speed. It can be clearly observed from Figure 23 that the proposed architecture attains high speed up over its software counterparts. In particular, when the number of training iterations reaches 1000, the CPU time of the proposed SOPC system is 733.14 ms. By contrast, the CPU time of Intel i7 is 1,0125.37 ms. The speedup of proposed architecture over the software counterpart is therefore 13.81.

The proposed architecture has superior speed performance over its software counterparts because there are limitations for exploiting the thread level parallelism. The GHA is an incremental training algorithm. Therefore, it is difficult to exploit parallelism among the computations for different training vectors. The inherent data dependency among different GHA stages (e.g., between principal component computation and weight vector updating) may slow down the computation speed due to costly data forwarding via shared memory. Moreover, the inputs (*i.e.*, x(*n*) and w_*j*_(*n*), *j* = 1,…,*p*) and outputs (*i.e.*, y(*n*), w*_j_*(*n* + 1), *j* = 1,…,*p*) of the algorithms are all vectors with large dimension. Large number of memory accesses required by GHA is another limiting factor for performance enhancement of software implementations. By contrast, the proposed architecture is able to perform data forwarding and memory accesses in an efficient manner. The employment of Buffers A, B and C allows the parallel operations of training vector fetching and weight vector computation. The latency for memory access can then be concealed. Moreover, the Buffers B and C are also designed for fast data forwarding between principal computation and weight vector updating without complicated memory management and external memory accesses.

In addition to having superior computational speed, the proposed architecture consumes lower power. Table 4 shows the power consumption of various GHA implementations. For the power estimation of GHA software implementations, the tool Joulemeter (developed by Microsoft Research) [24] is used. The tool is able to estimate the power consumed by CPU for a specific application. The power consumption of other parts of a computer such as main memory and monitor therefore can be excluded for comparisons. The power consumed by the proposed architecture is estimated by the PowerPlay Power Analyzer Tool [25] provided by Altera. From Table 4, it can be observed that the power consumption of the proposed architecture is only 0.4% of that of Intel I7 processor for GHA training (*i.e.*, 0.129 W*versus* 31.656 W). As compared with the low power multicore processor Intel i5 for laptop computers, the proposed architecture also has significantly lower power dissipation (*i.e.*, 0.129 W*versus* 1.292 W).

Table 5 compares the computation speed of various GHA architectures implemented by FPGA. Similar to Figure 23, the computation time of the proposed architecture is measured as the CPU time of the NIOS processor using the proposed architecture as the hardware accelerator. The clock rate of NIOS CPU in the system is 100 MHz. The vector dimension and the number of principal components associated with the proposed architecture are *m* = 16 × 16 and *p* = 16, respectively. The computation time of architectures in [18,19] with different *m* and/or *p* values are also included in the table.

Note that direct comparisons of these architectures may be difficult because the speed of these architectures are measured on different FPGA devices with different *m, p* and/or clock rates. To show the superiority of the proposed architecture, the comparisons are based on the same training size (*i.e.*, number of training vectors per iteration) and number of iterations. With larger vector dimension (*i.e.*, 16 × 16 *versus* 16 × 8), slower clock rate (*i.e.*, 100 MHz *versus* 136.243 M Hz), and the same number of principal components (*i.e., p* = 16), it can be observed from Table 5 that the proposed architecture still has faster computation speed as compared with the architecture in [19]. Although the architecture in [18] has fastest computation time, the architecture is suitable only for small vector dimension (*i.e., m* = 4 × 4) and small number of principal components (*i.e., p* = 4). All these facts demonstrate the effectiveness of the proposed architecture.

## 5. Concluding Remarks

Experimental results reveal that the proposed GHA architecture has superior speed performance over its software counterparts and other GHA architectures. With lower clock rate and higher vector dimension, the proposed architecture still has faster computation speed over the architecture in [19]. In addition, the architecture is able to attain higher CSR for texture classification as compared with other GHA architectures. In fact, all the CSRs are above 90% for all the experiments considered in this paper. The proposed architecture also has low area costs for fast PCA analysis with high vector dimension up to *m* = 32 × 32. The utilization of memory bits and embedded multipliers for FPGA implementation are independent of the vector dimension and the number of principal components. The proposed architecture therefore is an effective alternative for on-chip learning applications requiring low area costs, high classification success rate and high speed computation.

## Figures and Tables

**Table 1.** Performance analysis of various architectures for GHA training.

**Architectures**	**Adders**	**Multipliers**	**Registers**	**Latency**
proposed Architecture	*O*(*q*)	*O*(*q*)	*O*(*mp*)	*max*(*q*, 2*bp* + *s*)
[18]	*O*(*mp*)	*O*(*mp*)	*O*(*mp*)	*max*(*q* + 1,*p* + 1)
[19]	*O*(*p*)	*O*(*p*)	*O*(*mp*)	3*m*+*p*−1

**Table 2.** Hardware resource consumption of the proposed GHA architecture for vector dimensions *m* = 16 × 16 and *m* = 32 × 32.

*p*	**Proposed GHA with *m*** = **16** × **16**			**Proposed GHA with *m*** = **32** × **32**		
	
	**LEs**	**Memory Bits**	**Embedded Multipliers**	**LEs**	**Memory Bits**	**Embedded Multipliers**
3	35, 386/149, 760	0/6, 635, 520	704/720	85, 271/149, 760	7, 168/6, 635, 520	704/720
4	37, 731/149, 760	0/6, 635, 520	704/720	94, 244/149, 760	7, 168/6, 635, 520	704/720
5	40, 043/149, 760	7, 168/6, 635, 520	704/720	103, 394/149, 760	7, 168/6, 635, 520	704/720
6	42, 404/149, 760	7, 168/6, 635, 520	704/720	112, 679/149, 760	7, 168/6, 635, 520	704/720
7	44, 737/149, 760	7, 168/6, 635, 520	704/720	121, 940/149, 760	7, 168/6, 635, 520	704/720

**Table 3.** Hardware resource consumption of the SOPC system using proposed GHA architecture as hardware accelerator for vector dimensions *m* = 16 × 16 and *m* = 32 × 32.

*p*	**Proposed SOPC with *m*** = **16** × **16**				**Proposed SOPC with *m*** = **32** × **32**		
	
	**LEs**	**Memory Bits**	**Embedded Multipliers**	**LEs**	**Memory Bits**	**Embedded Multipliers**
3	44, 377/149, 760	446, 824/6, 635, 520	708/720	94, 736/149, 760	453, 992/6, 635, 520	708/720
4	46, 786/149, 760	446, 824/6, 635, 520	708/720	103, 968/149, 760	453, 992/6, 635, 520	708/720
5	49, 096/149, 760	453, 992/6, 635, 520	708/720	113, 207/149, 760	453, 992/6, 635, 520	708/720
6	51, 449/149, 760	453, 992/6, 635, 520	708/720	122, 537/149, 760	453, 992/6, 635, 520	708/720
7	54, 055/149, 760	453, 992/6, 635, 520	708/720	131, 779/149, 760	453, 992/6, 635, 520	708/720

**Table 4.** Power Consumption of Various GHA Implementations.

**GHA Implementations**	**Proposed Architecture**	**Multithreaded Software (16 threads)**	**Multithreaded Software (16 threads)**
Multicore Processor		Intel i7	Intel i5
FPGA Device	Altera Cyclone III EP3C120F780C8		
Clock rate	50 MHz	2.8 GHz	1.6 GHz
Estimated Power	0.129 W	31.656 W	1.292 W

**Table 5.** Computation Time of Various GHA Architectures.

**Architectures**	**Proposed Architecture**	[18]	[19]
Vector Dimension *m*	16 × 16	4 × 4	16 × 8
# of Principal Components *p*	16	4	16
FPGA Device	Altera Cyclone III EP3C120F780C8	Altera Cyclone III EP3C120F780C8	Xilinx Virtex 4 XC4VFX12
Clock Rate	100 MHz	75 MHz	136.243 MHz
Iteration Numbers	100	100	100
# of Training Vectors per Iteration	888 × 8	888 × 8	888 × 8
Computation Time	1.369 s	86.58 ms	2.09 s
